# Research on Differential Brain Networks before and after WM Training under Different Frequency Band Oscillations

**DOI:** 10.1155/2021/6628021

**Published:** 2021-03-20

**Authors:** Yin Tian, Huishu Zhou, Huiling Zhang, Tianhao Li

**Affiliations:** Bio-information College, Chongqing University of Posts and Telecommunications, Chongqing 400065, China

## Abstract

Previous studies have shown that different frequency band oscillations are associated with cognitive processing such as working memory (WM). Electroencephalogram (EEG) coherence and graph theory can be used to measure functional connections between different brain regions and information interaction between different clusters of neurons. At the same time, it was found that better cognitive performance of individuals indicated stronger small-world characteristics of resting-state WM networks. However, little is known about the neural synchronization of the retention stage during ongoing WM tasks (i.e., online WM) by training on the whole-brain network level. Therefore, combining EEG coherence and graph theory analysis, the present study examined the topological changes of WM networks before and after training based on the whole brain and constructed differential networks with different frequency band oscillations (i.e., theta, alpha, and beta). The results showed that after WM training, the subjects' WM networks had higher clustering coefficients and shorter optimal path lengths than before training during the retention period. Moreover, the increased synchronization of the frontal theta oscillations seemed to reflect the improved executive ability of WM and the more mature resource deployment; the enhanced alpha oscillatory synchronization in the frontoparietal and fronto-occipital regions may reflect the enhanced ability to suppress irrelevant information during the delay and pay attention to memory guidance; the enhanced beta oscillatory synchronization in the temporoparietal and frontoparietal regions may indicate active memory maintenance and preparation for memory-guided attention. The findings may add new evidence to understand the neural mechanisms of WM on the changes of network topological attributes in the task-related mode.

## 1. Introduction

Working memory (WM) is a system that maintains information online in order to complete a task or goal and carries out the operation and processing of the retained information. From the perspective of information processing, memory consists of three stages: encoding, retention, and extraction. The ability to retain information in WM is the basis for maintaining a good cognitive state [1]. Based on the retention stage during online WM, the WM behavioral performance can be predicted by spectral entropy [2], and the fusion features composed of spectral entropy and Lempel-Ziv complexity can effectively classify working memory load [3]. The central role of WM in human cognition, coupled with its limited capabilities, has led to current attempts to improve WM function through training. WM training may affect the functional connectivity between brain networks covered by the task. Kelly et al. suggest that widespread practice of WM tasks may lead to a permanent enhancement of resting-state frontoparietal connections [4]. Several functional magnetic resonance imaging (fMRI) studies have shown how WM training affected brain activity in a variety of ways, including interregional changes, anatomical changes, and functional connectivity changes [5]. However, fMRI with low temporal resolutions could not reveal the subsecond time precision required by the neural mechanism of integrating and coordinating processing between different functional regions during the online WM. These functions could be performed by oscillatory synchronization (i.e., correlation between neuronal activities of frequencies over a millisecond range of time), modulating interactions between neurons and regulating the communication of information between networks [6]. Studies have shown that different frequency band oscillations were associated with cognitive processing such as emotional memory in the brain [7, 8]. In terms of neurophysiology, oscillatory synchronization is where individual neurons fire regularly at the population oscillation frequency like clocks [9]. Oscillatory synchronization recorded by scalp EEG can be used to define interactions between different brain regions at relatively high temporal resolutions [10].

On the regional level, two common modes are utilized to study WM based on EEG. One is that one region of interest (ROI) or several ROIs are selected to study frequency band oscillations. For example, an intracranial EEG study showed that theta oscillations were a key mediator of WM, in which the theta power that changed in the dorsolateral prefrontal cortex (DLPFC), dorsal ACC (dACC), and hippocampus was associated with the ability to maintain information in WM [11]. During the reward-related WM, Kawasaki and Yamaguchi found that the amplitude of the prefrontal theta that increased during the retention period was positively correlated with the volume of visual working memory (VWM), indicating that it participated in the local synchronization of VWM. In addition, frontal beta oscillatory activities were identified as reward-related activities [12]. In a short-term memory study, Sauseng et al. found that the parietal alpha oscillations prevented external input from interfering with ongoing WM tasks [13]. Furthermore, neural synchronization of alpha oscillations inhibited information processing unrelated to WM [14]. Based on the local field potentials, Zhang et al. analyzed the dynamic characteristics of brain networks during WM. Experimental results showed that the cross-frequency coupling between theta and gamma increased with learning days, which played an important role in the WM [15]. That is, low-frequency neural oscillations can support information processing and interaction among a large range of neural clusters [16]. Transient or short-term frequency synchronization could be used to perform information interaction, information integration, and task coordination between different brain regions or different neural clusters in certain brain regions [17].

The other is that the relationship between various brain regions such as synchronization could be measured by EEG coherence for studying on activation of multiple brain regions [18]. EEG coherence was primarily a measure of phase consistency, suggesting a functional connection between paired brain regions. Strong coherence reflected the simultaneous oscillation of neurons, while weak coherence indicated independent activity between these neural clusters [19]. Using EEG coherence, Murias et al. found that some brain regions of the autistic population were too connected, while some brain regions were not connected enough [18]. Moreover, a study of EEG consistency in Alzheimer's disease revealed that alpha frequency band coherence in the temporo-parieto-occipital regions of the Alzheimer's group was significantly reduced [20]. In another study, EEG coherence results showed that the right hemisphere of the brain in the normal control group was more closely connected during the cognitive process, and the activation in the prefrontal and parietal regions was significantly higher than moderately depressed people [21]. In the WM task, the increased coherence of the theta frequency band in the frontal region and the decreased coherence of the high-alpha frequency band in the anterior region reflected the increased demand for central executive function in WM [22].

However, cognitive training interventions may affect not only changes in one or several regions but also changes in the integration and separation throughout the brain or brain subnetwork during online WM. Different neuroimaging methods, such as fMRI and transcranial magnetic stimulation (TMS), have shown that the frontal and parietal regions were engaged during online WM [23]. At the same time, theta, alpha, and beta oscillations were involved in the interaction between different brain areas, indicating the dynamic processing of information during the performance of WM tasks [24]. Early researchers believed that WM might be located in a specific area of the brain, but this view has been challenged. Recent studies have suggested that WM was not limited to a specific brain area because a single region would not be able to complete all memory storage and processing of individual life, work, and other experiences. WM as a complex cognitive system, which cannot be accomplished by reliance on a fixed neural pathway, involves interactions and integrations of millions of neurons in multiple brain regions.

The topology of the brain network quantified by the graph theory network index can distinguish the cognitive states within the individual and the cognitive abilities between individuals [25]. In neuropathology studies, the resting-state brain networks of depressed adolescents were significantly impaired compared to healthy controls with graph theory analysis [26]. Li and colleagues investigated the EEG network and clustering analyses of epilepsy in different frequency bands, reporting that the synchronization of the EEG network was enhanced during the preictal state which had higher network characteristics compared to the interictal state [27]. Yu et al. proposed that the local efficiency of cross-frequency coupling during epileptic seizures decreased significantly in the theta and alpha networks. [28]. Using EEG coherence analysis, another study found that the functional network of patients with mild depression deviated from the small-world network in terms of the shortest path length [21], indicating that the brain network construction based on the graph theory of EEG coherence was an effective way to distinguish between patients with mild depression and healthy patients. Based on graph theory coherence analysis, Langer et al. calculated the small-world properties of the resting-state EEG network before and after WM training. The results showed that better cognitive performance of WM indicated higher theta power and stronger small-world topology [23]. Another study found that synchronization was strengthened with increasing memory load among the frontoparietal regions known to underlie executive and attentional functions during memory retention [8]. According to the research of cognitive neuroplasticity during development, synchronization of oscillatory activities was an important indicator of cortical network maturation. In task-related oscillations involved in WM executive control, activation in the frontal and parietal regions was more pronounced and concentrated in adults than in children and adolescents [29]. Therefore, the synchronous changes of brain oscillations in the task execution stage by training may reveal the plasticity mechanism of WM.

Therefore, using the graph theory to construct the EEG WM brain network, we were able to study the transmission and integration of neural information during online WM. To our best knowledge, little is known about the neural pathways involved in the online WM before and after training. The continuous activity of the brain provided strong evidence that this activity reflected characterization during the retention period of WM. Many brain areas of the cortex and subcortex also exhibited similar sustained activity and formed a brain network for memory information interaction to support the processing and delivery of WM information [30]. We assumed that the effect of training on WM performance could be observed from the changes in neural oscillation activities before and after training. Thus, graph theory analysis is based on EEG coherence to construct the differential coherence networks before and after WM training. We first adopted the EEG coherence method to separately obtain the coherence matrix of theta, alpha, beta, and full frequency before and after WM training. Then, the changes of network attributes before and after WM training were studied by graph theory analysis. Consequently, the statistical differential networks of different frequency bands before and after training were obtained. In the present study, the plasticity of WM training was studied from the perspective of the whole-brain network, which may provide further support for the new concept of oscillatory action to understand WM training during online WM.

## 2. Materials and Methods

### 2.1. Subjects

Twenty right-handed normal male subjects (average age 21 years old) participated in the experiment. None had any cognitive impairment or history of mental and neurological diseases. The experiment was approved by the Ethics Committee of Chongqing University of Posts and Telecommunications. All subjects who participated in the experiment read the informed consent form in advance and signed it. After the experiment, subjects received corresponding compensation for their time and efforts.

### 2.2. Stimuli and Design

Subjects were asked to remain relaxed throughout the experiment and to suppress as far as possible any wide range of motion. They were asked to perform delayed WM tasks of three levels of difficulty (2, 4, and 8 items) over two sessions. The experimental content of the two sessions was the same, the only difference being that the subjects completed the first session without training and completed the second session after receiving short memory training.

The experimental procedure was stimulated, and the behavioral results were recorded by E-Prime (http://www.pstnet.com/eprime.cfm). A fixed cross (0.5° × 0.5°) was displayed at the center of the screen, and each session consisted of 60 trials (using memory load items at the three levels of difficulty). During the experiment, subjects were asked to limit blinking, eyeball rotations, and head movements and to respond correspondingly to the stimulus.

To begin, the fixed cross flashed for 50 ms, indicating that the experiment had started. Thereafter, the memory sequence was presented for 200 ms (the encoding period). The memory sequence consisted of a random composition of uppercase English letters from A to Z at three difficulty levels (easy: 2 items, medium: 4 items, and difficult: 8 items). They appeared randomly with the same probability. Then, after a 3000 ms delayed interval (the retention period), the test sequence (one English letter) was presented as a probe item on the screen for 100 ms. At this point, subjects were required to judge whether the test sequence had appeared in the previous memory sequence. If the probe item had not appeared in the previous memory sequence, subjects pressed the “F” key with their left index finger. If it had, subjects pressed the “J” key with their right index finger.

### 2.3. EEG Recordings and Preprocessing

A 64-channel NeuroScan system was used to record subjects' EEG data (Quik-Cap, bandpass filtering: 0.05-100 Hz; sampling rate: 1000 Hz; and impedance less than 5 k*Ω*), and the Cz electrode was taken as the reference electrode for online EEG acquisition.

The offline processing of the EEG data mainly included rereferencing, data segmentation, artifact removal, filtering, and baseline correction. Rereferencing means the EEG recordings were infinitely rereferenced by using reference electrode normalization techniques [31, 32]; segmentation means EEG data was extracted from 100 ms before the onset of the memory sequence to 100 ms after the subjects' response onset; artifact removal means electronystagmogram (EOG) and myoelectricity (EMG) were eliminated by a blind source separation technique. At the same time, the data segments with a voltage amplitude range exceeding ±100 *μ*V were removed. The EEG recordings were then filtered with a bandpass of 0.5-45 Hz. The data was baseline corrected using the 100 ms before the memory array onset. After the above preprocessing, each subject's EEG data had 120 trials (60 trials for each session). Finally, the retention period (3000 ms) for each trial was extracted for subsequent analysis.

### 2.4. Behavioral Scores

When the subjects carried out the reaction time and reaction accuracy tasks, they either sacrificed the accuracy rate in exchange for the reaction rate or sacrificed speed to obtain high reaction accuracy [33]. In the delayed WM matching task, the speed-accuracy trade-off problem arose. Therefore, we used signed residual time (SRT) for the subject's performance score [34]. SRT was then used to measure the balance between speed and accuracy of the subjects when they were performing the task. The SRT scoring rules were defined as follows:

(1) $$\begin{array}{c} \underset{i}{\sum }\left(2{\text{RACC}}_{i}-1\right)\left(\text{MT}-T_{i}\right), \end{array}$$

where *i* represented the total number of trials, indicating the reaction accuracy of a single trial (RACC: 0 indicated reaction error, 1 indicated correct response), and MT indicated the maximum allowable reaction time for the *i*-th trial. *T*_*i*_ indicated the latency of the reaction. The total SRT score was the sum of each trial score. In other words, for the trial of the correct response, the remaining time was added when the total SRT score was calculated, and the remaining time was subtracted for the incorrect reaction trial. Through the above process, each subject received an SRT score.

### 2.5. Coherence Matrix Construction

The coherence of the EEG signals reflected the correlation of the time domain signals of the two brain regions in different frequency bands. The coherence function between the two signals *a* and *b* was calculated as follows:

(2) $$\begin{array}{c} C^{2}\left(f\right)=\frac{{\left|Pab\left(f\right)\right|}^{2}}{\left[P_{aa}\left(f\right)P_{bb}\left(f\right)\right]}, \end{array}$$

where *f* was the frequency of the EEG signals; *P*_*aa*_(*f*) and *P*_*bb*_(*f*) denoted the auto power spectrum of the signals *a* and *b*, respectively; and |*Pab*(*f*)| represented the modulus of the cross-power spectrum between the signals *a* and *b*. *C*(*f*) was the coherence coefficient, indicating the basic characteristics of the EEG signal at the two electrodes (such as consistency of the EEG amplitude, frequency, and phase). The coherence coefficient ranged from 0 to 1. The coefficient value with 0 indicated that the two signals *a* and *b* were absolutely not synchronized; that is, they were completely independent [22, 30]. The coherence coefficient equal to 1 meant that the two signals *a* and *b* were synchronized, and the coherence coefficient with the value between 0 and 1 meant that there was partial coherence [22, 30].

**Figure 1:**
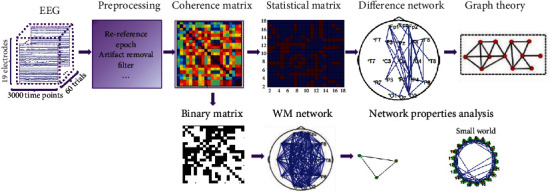
Construction process of coherence and differential networks.

Figure 1 shows the process flow of the coherence network and the differential network based on a paired *t*-test. First, to reduce the volume conductor effect to a minimum, 19 electrodes were selected as nodes of the WM brain network. Then, power spectral densities in theta (4-7 Hz), alpha (8-12 Hz), and beta (13-30 Hz) frequency bands were extracted by using Welch's method. After that, coherence was used to measure the relationship between any two of the 19 electrodes. In other words, the coherence coefficient represented the strength of the relationship (i.e., functional connection) between paired nodes and was taken to represent the edges of the brain networks before and after WM training. The coherence matrix was a 19 × 19 symmetric matrix. For the coherence matrix of the full frequency band before and after training, an appropriate threshold was selected for binarization. To obtain a differential matrix induced by WM training in theta, alpha, and beta frequency bands, a paired *t*-test was used to analyze statistically the corresponding elements (i.e., coherence values) in the coherence matrix of each subject before and after the training. Finally, the statistical network (binary network) before and after the WM training was obtained to examine whether the subjects' coherence coefficient between two nodes before and after training was significantly different (*p* < 0.05, FDR correction).

### 2.6. Network Properties

The graphic analysis was used to construct the brain networks before and after WM training, and the network topological properties were measured by the optimal path length (*L*_p_), clustering coefficient (CC), local efficiency (*E*_loc_), global efficiency (*E*_g_), degree (Deg), and small-world properties of the network [35–37]. The network properties were defined as shown in Table 1 below.

## 3. Results

### 3.1. Behavioral Analysis

**Figure 2:**
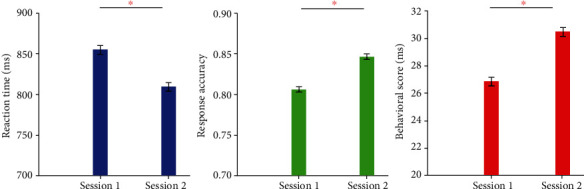
Behavioral analysis before and after WM training. Session 1: before training. Session 2: after training. Red star means significance (*p* < 0.05).

Figure 2 shows the behavioral analysis of the subjects before and after WM training. According to the statistical results from a paired *t*-test, the average reaction time in session 1 was significantly greater than that in session 2 (*t* = 2.744, *p* < 0.05), while the response accuracy (*t* = 2.716, *p* < 0.05) and SRT scores were significantly higher in session 2 than session 1 (*t* = 4.750, *p* < 0.001).

### 3.2. Network Property Analysis

**Figure 3:**
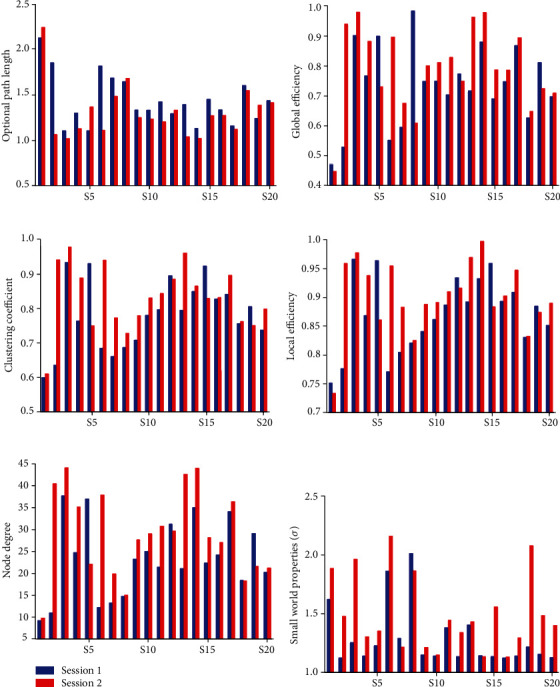
Analysis of WM network properties before and after training with each subject. Session 1: before training. Session 2: after training.

First, the appropriate threshold was selected to binarize the coherence matrix before and after training during the retention period of WM. To ensure connectivity between the nodes of the WM brain networks, the threshold selection principle was the maximum threshold without isolated nodes. If the coherence coefficient value was less than the given threshold, the binary matrix element was 0, indicating that the correlation or connection between the two nodes was weak; that is, the information interaction between the brain nodes was not frequent; if the coherence coefficient value was greater than or equal to the given threshold, the element of the matrix was 1, indicating that the correlation or connection between the two electrode signals was strong; that is, the information interaction between the brain nodes was close.  Figure 3 illustrates the changes in WM coherence network properties before and after training.

In Figure 3(a), 16 out of 20 subjects' optimal path lengths in their WM brain coherence networks were obviously shorter after training (session 2). Moreover, the statistical result of the paired *t*-test showed that the optimal path lengths in the WM networks between session 2 and session 1 were significantly different (*t* = 2.304, *p* < 0.05), and for 20 subjects, the averaged path length of the coherence network in session 2 was shorter than that in session 1. As shown in Figure 3(b), 15 out of 20 subjects' global efficiencies in their WM coherence networks in session 2 were lower than those in session 1; the results of statistical tests revealed that the global efficiencies in the WM networks between session 2 and session 1 were significantly different (*t* = 1.515, *p* < 0.05), and the 20 subjects' averaged global efficiency in the WM networks in session 2 was higher than that in session 1.

In Figure 3(c), 16 out of 20 subjects' clustering coefficients of their WM networks increased after training (session 2); the result of a paired *t*-test showed that the clustering coefficient of the delayed WM network between session 2 and session 1 was obviously different (*t* = 2.114, *p* < 0.05), and the averaged clustering coefficient of 20 subjects' WM networks in session 2 was larger than that in session 1.  Figure 3(d) shows that 16 out of 20 subjects' local efficiencies of the WM networks in session 2 were higher than those in session 1. The results of a paired *t*-test indicated that there was an obvious difference in the local efficiency of the delayed coherence networks between session 2 and session 1 (*t* = 2.035, *p* < 0.05), and the 20 subjects' averaged local efficiency of WM networks in session 2 was greater than that in session 1.

Figure 3(e) illustrates that 16 out of 20 subjects' degrees of WM networks in session 2 were higher than those in session 1; the results of a *t*-test showed that there was an obvious difference in the degree of WM networks between session 2 and session 1 (*t* = 2.502, *p* < 0.05), and the 20 subjects' averaged degree of WM networks in session 2 was greater than that in session 1. From Figure 3(f), it can be seen that the total WM network of all 20 subjects had small-world properties (*σ* ≫ 1), and 17 out of 20 subjects' WM network small-world properties in session 2 were stronger than those in session 1; the result of a paired *t*-test showed that there was an obvious difference in the small-world properties of WM networks between session 2 and session 1 (*t* = 3.672, *p* < 0.05), and the 20 subjects' averaged small-world property in session 2 was stronger than that in session 1.

### 3.3. Difference Statistical Networks

**Figure 4:**
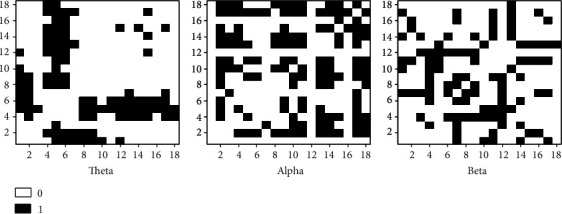
Coherence statistical matrix.

**Figure 5:**
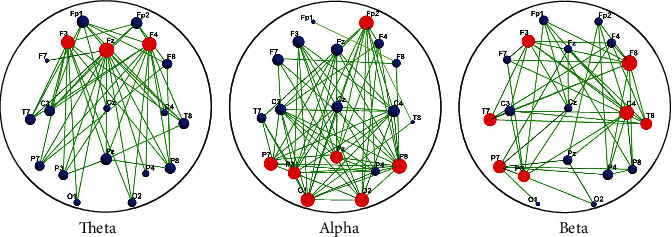
Difference statistical networks. Red nodes represent the hub nodes. Blue nodes represent the nonhub nodes. Node size reflects the degree.

The paired *t*-test was utilized to obtain statistically the corresponding elements of the coherence matrixes for the theta, alpha, and beta frequency band oscillations before and after the training. That is, if there was a significant difference in the coherence values of 20 subjects' WM networks between the two nodes before and after WM training (*p* < 0.05, FDR correction), the corresponding element of the statistical matrix was set to 1, and if there was no significant difference, the element of the statistical matrix was set to 0. Finally, the statistical (binary) matrixes of theta, alpha, and beta frequency bands before and after WM training were obtained; thereby, the differential networks of theta, alpha, and beta frequency bands were acquired. Figures 4 and 5 show the coherence statistical matrixes and differential networks of the theta, alpha, and beta frequency band oscillations before and after the WM training.

Based on graph theory analysis, we knew that a network consisted of many nodes (or regions of interest) and edges between paired nodes, among which some nodes played the role of a central hub. If these nodes were destroyed, the entire network would not be able to process and transmit information correctly, so these nodes were considered to be hub nodes of the network. The hub nodes reflected some of the important brain regions in the structural or functional brain network, interacting with functional brain regions to perform cognitive tasks effectively [38].

Node degree and betweenness were the most commonly used methods to define the hub nodes of the differential networks before and after WM training [39]. The present study utilized node degrees to define the hub nodes of the differential networks before and after WM training.  Table 2 displays the node degrees of the 19 electrodes of the differential networks before and after WM training in the three frequency bands oscillations (theta, alpha, and beta).

From the statistical results, for the differential network of the theta frequency band oscillations, the F3, Fz, and F4 electrodes were the hub nodes, with degrees of 10, 15, and 12, respectively. For the differential network of the alpha frequency band oscillations, the Fp2, P7, P3, Pz, P8, O1, and O2 electrodes were the hub nodes, with degrees of 13, 11, 9, 8, 15, 12, and 12, respectively. For the beta frequency band frequency differential network, the F3, F8, T7, C4, T8, P7, and P3 electrodes were the hub nodes, with degrees of 8, 10, 8, 9, 7, 8, and 7, respectively.

## 4. Discussion

The behavioral results showed that the indexes (ACC, RT, and SRT) of the online WM after training were significantly higher than those before training. EEG analysis based on the network level indicated that the online WM network had higher clustering coefficients and shorter optimal path lengths on the full frequency after training. For the theta network, there was an increased synchronization among nodes in the frontal region after training. For the alpha network, the long-distance information interaction in the fronto-occipital and frontoparietal lobes increased after training. For the beta network, synchronization between frontoparietal and temporoparietal mainly increased after training. These results showed that both behavioral performance and network function were improved by WM training.

### 4.1. Behavioral Performance during Online WM before and after Training

The results of behavioral statistical analysis in Figure 2 showed that the average response speed of the subjects after training was faster and the average response accuracy of the subjects after training was significantly higher than those before training. Moreover, SRT was significantly higher than the pretraining behavioral scores, which suggested the consistent conclusion that short-term WM training could improve individual WM behavioral performance [40–42].

### 4.2. Full Frequency Band Networks before and after Training

In the present study, the relationship between two nodes of the WM network was measured by EEG coherence, and the different changes of network attributes before and after training were obtained by graph theory analysis (Figure 3).

The statistical results in Figures 3(a) and 3(b) revealed there was a significant difference in the optimal path length and global efficiency of the WM network before and after training. That is, the optimal path length of the network after training was lower than that before training, indicating that the information transmission efficiency of the trained WM network was more efficient. Global efficiency after training was significantly higher than that before training, illustrating the global information that the global information transmission capability was stronger. The statistical results in Figures 3(c) and 3(d) confirmed that 20 subjects' clustering coefficients and local efficiencies were superior to those without training, which presented the enhanced brain network connectivity, the tighter information interaction between network nodes, and the more intensive local information transmission capability of trained WM networks. A denser connection of the brain network and a shorter optimal path length after training may ensure efficient processing and transmission of WM information. From the statistics in Figure 3(e), it can be noted that the node degree of the memory network after training was significantly larger than that of the pretraining memory networks, which indicated that there were more connections and more frequent information interactions between nodes in the memory training networks. Figure 3(f) illustrates that both the trained and untrained WM networks represented small-world characteristics. That is, compared with a random network, the WM brain networks during the retention period had a higher clustering coefficient and a similar optimal path length. The statistical results showed that the small-world properties (*σ*) of the networks after training were stronger than those before training, which indicated that the networks efficiently completed the processing and delivery of WM information at a relatively low connection cost.

Previous studies have shown that resting-state networks after WM training significantly reduced clustering coefficients and normalized the shortest path lengths [43]. This was contrary to our conclusion in the task state, which supported the reduced shortest path length and the increased clustering coefficient. The inconsistency may be due to the spatial *N*-back experimental paradigm in the previous study different from the digital delayed match paradigm in the present study. One study showed that higher global network integration and modularity predicted significantly better performance in visual-spatial WM, while numerical WM was superior in subjects with highly clustered brain networks [44]. In the present study, the digital WM training was adopted, and the higher local efficiency in the task execution after the training further proved that the ability of digital WM improved after training. Or it may be due to the different brain network patterns between the resting and task states. We focused on the changes of topological attributes in the task state before and after the training rather than in the resting state with multiple training.

### 4.3. Three Frequency Band Differential Networks before and after Training

From the discussion above, individual WM may be improved by training. On the whole, the properties of trained WM networks were superior to the network properties of untrained WM networks, suggesting that individual WM was plastic.

A previous study has shown that different frequency band oscillations involved information exchange and cognitive processing between brain regions [45]. Memory was thought to depend mainly on the degree of synchronization between neurons, which was particularly important in WM. Typically, alpha, beta, and theta oscillations were involved in memory processes. To explore the dynamical changes on the brain network by WM training, the present study constructed the differential matrixes (Figure 4) and the differential networks (Figure 5) of theta, alpha, and beta frequency bands before and after training based on the retention period during online WM.

Consistent with the previous report [46, 47], our results showed that the frontal theta enhancement was observed in the online WM after training (Figure 5). The synchronization of theta oscillations helped coordinate neural communication between multiple brain regions, which was beneficial to maintain short-term memory. For the theta network in the present study, the node degrees at the F3, Fz, and F4 electrodes increased after WM training, pointing to increased coherence between frontal nodes and occipital-parietal nodes under the theta frequency band after WM training. The previous studies based on the region levels found the theta frequency band to be enhanced in the different regions. For example, the previous study suggested that the theta oscillations in parietal coupling were a marker of central executive function in WM [22, 48]. In addition, prioritizing of information in WM was associated with theta oscillations in the lateral prefrontal cortex [49]. The frontal theta enhancement was primarily evident in high performers on the order WM task. In elucidating the role of the theta frequency band in WM maintenance, Tóth et al. suggested that the theta oscillations in the midfrontal were a possible basis for the active maintenance process particularly susceptible to the effects of aging [50]. In general, it was widely accepted that the frontal theta oscillations were mainly related to the executive function of WM [51–53]. Some studies speculated that the prefrontal theta activity seemed to reflect general task requirements, such as attention resource deployment during WM [54]. However, the disagreement could be due to the fact that related research studies are limited on the regional level. Thus, the present results based on the whole-brain network level suggested that the increased synchronization of the frontal theta oscillations in the retention period seemed to reflect the enhanced executive ability of WM by training and the more mature resource deployment after training.

For the alpha network, the size of node degrees at the frontal (Fp2), parietal (P7, P3, Pz, and P8), and occipital (O1 and O2) electrodes was significantly bigger after training than that before training, indicating increased coherence between paired nodes in the fronto-occipital network under the alpha frequency band after WM training. Moreover, it also involved the long-range integration of “top-down” information processing between the frontoparietal brain regions. This was in line with previous research. Previous research found that alpha oscillations have also been shown to be particularly important in top-down visual processing and visual attention [55]. In an experiment investigating imaginary instrumental performance, researchers discovered that the characteristic coherence mode of the alpha frequency band mainly involved the activation of “top-down” information processing. Here, the input to the sensory region came from multiple cortical areas. Thus, top-down information processing could be considered a distillation of all transient activation in the cortex, where a guide to future actions was generated for the selection of new sensory inputs [45]. In addition, alpha oscillations in the occipital-parietal cortex were associated with suppression-independent information [56, 57], and the enhanced alpha oscillations were associated with suppression of sensory field inputs to protect internal attention from external interference during the WM retention period [58]. Therefore, the increased alpha oscillatory synchronization in the frontoparietal and fronto-occipital regions by training in the present study may reflect the enhanced ability to suppress irrelevant information during the delay and pay attention to memory guidance after training.

We found that the node degrees of the beta network at the frontal (F3, F8), temporal (T7, C4, and T8), and parietal (P3, P7) electrodes were significantly increased by WM training, indicating increased coherence between paired nodes in the temporoparietal and frontoparietal regions under the beta frequency band after WM training. Stein et al. reported that the synchronization between the temporal and parietal regions of the lower-beta frequency band increased during multimodal semantic processing. And their results found synchronized activation in the frontal and posterior parietal cortex during the WM retention period [59]. Moreover, Daume et al. proposed the enhanced beta oscillatory synchronization from the medial temporal lobe (MTL) to the temporal-occipital visual region during the WM retention period [60]. Their results were consistent with our findings based on the whole-brain network level. Earlier studies linked the beta oscillatory synchronization between the temporal-occipital regions with the WM retention period [61]. As long as the current cognitive state was maintained, the beta oscillatory synchronization could be improved [62]. Further research suggested that the effect of the beta oscillatory synchronization between the MTL and the postaudiovisual region (including the superior temporal sulcus (STS) and lateral occipital complex (LOC)) emphasized beta oscillations as a central role in maintaining visual object representation in the ventral flow [60]. More than that, higher beta power was associated with longer memory duration [63]. In addition, previous studies reported an intimate causal relationship between prefrontal beta oscillatory desynchronization and memory formation [64]. Therefore, the enhanced beta oscillations in the temporoparietal and frontoparietal regions after training may indicate active memory maintenance and preparation for memory-guided attention during the WM retention period.

## 5. Conclusion

The present study combined EEG coherence and graph theory analysis and examined the topological changes by WM training based on the whole-brain network. We have provided a new concept of oscillatory activities to understand WM training during the retention period of online WM from the perspective of the whole-brain network. Overall, frequency band oscillatory synchronization was related to the specific cognitive mechanism during the retention period. Future work can explore whether more effective training effects can be obtained by adjusting frequency band oscillatory synchronization in WM training.

## Figures and Tables

**Table 1:** Brain network properties.

Measure	Binary definition	Meaning
Optimal path length (*L*_p_)	*L*_p_ = 1/*N*(*N* − 1)∑_*a*,*b*∈*V*,*a*≠*b*_*L*_*ab*_	*L*_p_: optimal path length of the WM brain network *L*_*ab*_: shortest path for information transfer between nodes *a* and *b*. Through *L*_*ab*_, information can be transferred more quickly and effectively *V*: collection of all the nodes in the WM network *N*: number of network nodes
Global efficiency (*E*_g_)	*E*_g_ = 1/*N*(*N* − 1)∑_*a*,*b*∈*V*,*a*≠*b*_(1/*L*_*ab*_)	*E*_g_: global efficiency; the extension of the optimal path length of networks. Can measure the global information transmission capacity of networks
Clustering coefficient (CC)	*C*_*a*_ = 2*e*_*a*_/*k*_*a*_(*k*_*a*_ − 1) CC = 1/*N*∑_*a*∈*V*_*C*_*a*_	*C*_*a*_: clustering coefficient of node *a*; describes the connectivity level between node *a* and its neighbor nodes *V*_*a*_: subgraph formed by all the neighbors of node *a* *e*_*a*_: actual number of edges in the subgraph *V*_*a*_ CC: clustering coefficient of the network
Local efficiency (*E*_loc_)	*E*_loc_*a*__ = 1/*N*_*V*_*a*__(*N*_*V*_*a*__ − 1)∑_*b*,*c*∈*V*_*a*_,*c*≠*b*_(1/*L*_*bc*_) *E*_loc_ = 1/*N*∑_*a*∈*V*_*E*_loc_*a*__	*E*_loc_*a*__: local efficiency of node *a* represents the information exchange ability after deleting node *a* in subgraph *V*_*a*_ *L*_*bc*_: shortest path between nodes *b* and *c* in the subnetwork *V*_*a*_ *E*_loc_: local efficiency of the network
Degree (Deg)	Deg = 1/*N*∑_*a*∈*V*_Deg_*a*_	Deg*_a_*: degree of node *a* Deg: degree of the network
Small-world properties	*γ* = CC^real^/CC^random^ > 1 *λ* = *L*_p_^real^/*L*_p_^random^ ≈ 1 *σ* = *γ*/*λ*	CC^real^: clustering coefficient of the real network CC^random^: clustering coefficient of the random network *L*_p_^real^: optimal path length of the real network *L*_p_^random^: optimal path length of the random network *σ*: small-world properties; when *σ* is significantly larger than 1, the network satisfies small-world properties

**Table 2:** Node degrees of the differential networks.

Node degree	Theta	Alpha	Beta
Fp1	7	1	5
Fp2	6	*13*	4
F7	1	6	4
F3	*10*	*7*	*8*
Fz	*15*	*7*	4
F4	*12*	4	5
F8	4	3	*10*
T7	5	4	*8*
C3	5	6	6
Cz	2	6	4
C4	2	7	*9*
T8	5	1	*7*
P7	4	*11*	*8*
P3	4	*9*	*7*
Pz	6	*8*	5
P4	2	3	6
P8	5	*15*	5
O1	2	*12*	1
O2	3	*12*	2

## Data Availability

The simulation and analysis data used to support the findings of this study are available from the corresponding author upon request.
